# ﻿Four new species of *Entoloma* subgenus *Cyanula* (Entolomataceae, Agaricales) from subtropical regions of China

**DOI:** 10.3897/mycokeys.116.145568

**Published:** 2025-04-29

**Authors:** Lin-Gen Chen, Hong Chen, Ling Ding, Yu-Qin Xu, Hui Zeng, Sheng-Nan Wang, Jun-Qing Yan

**Affiliations:** 1 Jiangxi Provincial Key Laboratory of Excavation and Utilization of Agricultural Microorganisms, Jiangxi Agricultural University, Nanchang 330045, China; 2 Institute of Edible mushroom, Fujian Academy of Agricultural Sciences, Fuzhou 350011, China; 3 Jiangxi Provincial Key Laboratory of Subtropical Forest Resource Cultivation, College of Forestry, Jiangxi Agricultural University, Nanchang 330045, China

**Keywords:** Basidiomycetes, new taxa, phylogeny, taxonomy

## Abstract

In this study, four species of EntolomasubgenusCyanula (*E.orientosinense*, *E.subgriseosquamulosum*, *E.subpraegracile*, and *E.wuyishanense*) from subtropical regions of China, are described as new to science based on morphological and phylogenetic analyses. Morphologically, *E.orientosinense* is characterized by the white basidiomata, relatively large basidiospores, and carneogriseum-type lamellae edge; *E.subgriseosquamulosum* is recognized by the fuscous pileus, crowded and adnate lamellae, and medium-sized basidiospores; *E.subpraegracile* is identified by the yellow pileus and intervenose lamellae with sterile or heterogeneous edge; *E.wuyishanense* is distinct by the blue basidiomata and fertile lamellae edge with slightly bluish pigmentation near the stipe. *Entolomaorientosinense* belongs to sect. Caesiocincta, subsect. Queletia, and *E.wuyishanense* belongs to sect. Poliopodes. The remaining two species each form independent branches and do not belong to any known sections. Detailed descriptions, color photos, and a key to related species are presented.

## ﻿Introduction

*Entoloma* (Fr.) P. Kumm. is one of the most diverse genera within Agaricales, well-characterized by pink to brownish spore prints and angular basidiospores viewed in all views (Co-David et al. 2009). It shows extremely wide geographical distribution, occurring from the frigid zone to the tropics, and from alpine to basins, with most members being saprobic on shady and humid soil, mosses, or rotten wood in forests (Horak 1980; Noordeloos 1981; Largent 1994; Reschke et al. 2022b). So far, approximately 2000 *Entoloma* species have been reported in the world. In China, however, there are relatively few reports about the species of this genus, with approximately 200. Among them, some of the newly discovered species published earlier were not classified into specific subgenera (Bi et al. 1986; Zhang et al. 1994; He et al. 2011).

In the past, based on morphological taxonomy, *Cyanuli* was introduced by Romagnesi (1974) as a section within *Rhodophyllus* Quél (= *Entoloma*). The combination EntolomasectionCyanula (Romagn.) Noordeloos belonged to EntolomasubgenusLeptonia of wide sense. However, the subg. Leptonia, traditionally divided into three sections, viz. *Leptonia*, *Cyanula*, and *Griseorubida* (Noordeloos 2004), turned out to be polyphyletic. Sect. Leptonia of belonged to the /*Nolanea*-*Claudopus* clade, and *Cyanula* and *Griseorubida* to the /*Inocephalus*-*Cyanula* clade (Co-David et al. 2009). Morphologically, species of sect. Leptonia exhibited clamp connections, whereas species of *Cyanula* lacked clamp connections. Based on these, sect. Cyanula was elevated to subgenus rank (Noordeloos and Gates 2012; Noordeloos et al. 2022a).

The species of Entolomasubg.Cyanula are mainly characterized by their collybioid habit, vividly colorful (often blue, violaceous to brown) and squamulose pileus, absence of clamp connections, and presence of brilliant granules and intracellular pigments in hyphae. So far, at least 500–600 species of E.subg.Cyanula have been discovered worldwide.

According to previous studies, there are 13 species belonging to Entolomasubg.Cyanula in China, 7 of which were newly described (He et al. 2011, 2012; He et al. 2017). In the past few years, during our surveys on the diversity of macrofungi in the subtropical regions of China, we have found that the species diversity under *E. subg. Cyanula* is extremely rich. In this study, four species of this subgenus are newly described based on morphological comparisons and phylogenetic analyses.

## ﻿Materials and methods

### ﻿Morphological studies

The collection sites of the specimens in this study were all located in the subtropical region of East China, and these specimens were deposited in the Herbarium of Fungi, Jiangxi Agricultural University (HFJAU). Fresh specimens were photographed in the field and macroscopically recorded. The color notations followed the Methuen Handbook of Colour (Kornerup and Wanscher 1978). Microscopic structures were studied under an Olympus BX53 microscope (Olympus corporation, Tokyo, Japan) by making squash preparations of sections of dried specimens. The sections were hydrated with 5% KOH solution or H_2_O, and 1% Congo red was used as the staining agent when observing colorless tissues. Melzer’s reagent was selected for determining whether the spores were amyloid or not (Horak 2005). At least 20 basidiospores, basidia, and cystidia were measured for each collection. The range of spore size is expressed as the form (a) b–c (d), in which “a” and “d” represent the minimum and maximum values, and 90% of the spores falling within the range “b–c”. The meanings of the other spore characteristics were as follows: “Q” stood for the ratio of length and width; “av” symbolized average value; “n” meant the number of measurements; and “Qm” indicated average “Q” ± standard deviation (Bas 1969). The morphological descriptions were based on the work of Noordeloos et al. (2022a).

### ﻿DNA extraction, PCR amplification, and sequencing

Genomic DNA was extracted from dried specimens with the NuClean Plant Genomic DNA kit (CWBIO, China) (Wang et al. 2022). The nrDNA ITS and LSU regions were amplified respectively using the primer pairs of ITS1F/ITS4, LR0R/LR5 (White et al. 1990).

PCR amplification was conducted with a 25 μL reaction system as follows: 1 µL DNA, 1 µL each for forward and reverse primers, 9.5 µL ddH_2_O, and 12.5 µL 2 × Taq Master Mix (Dye Plus, Vazyme Biotechnology Co. Ltd., Nanjing City, China). PCR was carried out using a touchdown amplification procedure following Chen et al. (2024). The PCR products were sequenced by Qing Ke Biotechnology Co. Ltd. (Wuhan City, China).

### ﻿Alignment and phylogenetic analyses

In total, 173 sequences (126 ITS sequences and 47 LSU sequences) of 126 samples were used for phylogenetic analyses based on Bayesian inference (BI) and Maximum likelihood (ML). The selection of sequences for the phylogenetic analyses was based on the results of ITS BLAST and of Noordeloos et al. (2022a) (Table 1). Some species of Entolomasubg.Nolanea were designated as outgroups. The ITS sequences and the LSU sequences were separately aligned on the MAFFT online server using the automatic selection of algorithm (Katoh et al. 2019). First, phylogenetic trees were constructed separately for ITS and LSU and their congruence was checked. BI and ML phylogenetic analyses of the concatenated sequences were run using MRBAYES v.3.2.7a (Ronquist et al. 2012) and IQTREE v.2.1.2 (Nguyen et al. 2015), respectively. For the ML analysis, models of sequence evolution were assessed in IQ-Tree prior to the analysis and allowing the partitions of sequences to have different seeds (-spp) and the results were the following: TPM2 + F + I + G4 for ITS and HKY + F + I + G4 for LSU. Ultrafast bootstrap support values were calculated from 1000 replicates. For the BI analysis, the best-fit models were determined by PARTITIONFINDER (Zhang et al. 2020) based on Bayesian information criterion (BIC) and the results were the following: GTR + F + I + G4 for ITS and HKY + F + I + G4 for LSU. The Monte Carlo Markov chains were run for 40 million generations. The first 25% of trees were discarded as burn-in. The nodes with Bayesian posterior probabilities (BI-PP) ≥ 0.95 and ML bootstrap proportions (ML-BP) ≥ 95% were considered as statistically supported. A nexus file containing sequence alignment and the original trees of ML and BI analyses are provided in Suppl. material 1.

**Table 1.** Details of sequences used in the phylogenetic analyses. Newly generated sequences were in bold.

Species	Location	Voucher Number	GenBank No. (ITS)	GenBank No. (LSU)	Sequence origin
*Entolomaalbidosimulans*	Australia	MEN 2004-065, isotype	—	MK277956	Varga et al. (2019)
*E.albinellum*	USA	TENN:070403	KY777375	—	Unpublished in GenBank
*E.argus*	Vietnam	LE F-312694, holotype	OM987263	OM996175	Morozova et al. (2022)
*E.argus*	Vietnam	LE F-315916	OM987264	—	Morozova et al. (2022)
*E.arion*	Vietnam	LE F-312691, holotype	OM987259	OM996176	Morozova et al. (2022)
*E.arion*	Vietnam	LE F-312692	OM987260	—	Morozova et al. (2022)
*E.arion*	Vietnam	LE F-315917	OM987261	—	Morozova et al. (2022)
*E.asprellum*	Estonia	TUF106064	UDB011486	—	UNITE
*E.atropapillatum*	Brazil	FK0898, holotype	KF679354	KF738940	Karstedt et al. (2020)
*E.azureosquamulosum*	China	HKAS53408	JQ410334	JQ410326	He et al. (2012)
*E.azureosquamulosum*	China	GDGM29254	JQ410335	—	He et al. (2012)
*E.azureosquamulosum*	China	GDGM27355, holotype	NR_137086	NG_059214	He et al. (2012)
*E.caespitosum*	China	GDGM27564	JQ281477	JQ320130	He et al. (2012)
*E.caespitosum*	China	GDGM24025	JQ281490	JQ410327	He et al. (2012)
*E.caespitosum*	China	GDGM24026	JQ281491	JQ320133	He et al. (2012)
*E.calceus*	Norway	O-F-259457, holotype	NR_182489	—	Noordeloos et al. (2022b)
*E.calceus*	France	LIP0402265	ON008492	—	Noordeloos et al. (2022b)
*E.calceus*	Norway	JL12-19	ON008493	—	Noordeloos et al. (2022b)
*E.callipygmaeum*	Russia	LE312488	MZ145205	—	Dima et al. (2021)
*E.callipygmaeum*	Russia	LE312487	MZ145206	—	Dima et al. (2021)
*E.callipygmaeum*	Russia	LE253784, holotype	MZ145207	—	Dima et al. (2021)
*E.carneogriseum*	Norway	O-F-256479	UDB07673714	—	UNITE
*E.cetratum*	Sweden	LE311888, neotype	OL338280	—	Reschke et al. (2022a)
*E.chalybeum*	Russia	LE254353	KC898445	KC898500	Morozova et al. (2014)
*E.chalybeum*	Denmark	TUF105760	UDB034191	—	UNITE
*E.consanguineum*	New Zealand	PDD80751	MW775252	—	Unpublished in GenBank
*E.consanguineum*	New Zealand	PDD80751	MW775268	—	Unpublished in GenBank
*E.coracis*	Norway	O-F-256850, holotype	MW934571	MW934251	Crous et al. (2021b)
*E.coracis*	Norway	O-F-67255	MW934572	—	Crous et al. (2021b)
*E.coracis*	Norway	O-F-251952	MW934573	—	Crous et al. (2021b)
*E.corvinum*	France	FA4261	OR419868	—	Armada et al. (2023)
*E.cyanostipitum*	China	GDGM31318, holotype	KY711237	KY972694	He et al. (2017)
*E.cyanostipitum*	China	SAAS2239	KY711238	KY972695	He et al. (2017)
*E.cyanostipitum*	China	GDGM31294	KY972700	KY972693	He et al. (2017)
*E.dislocatum*	Spain	L0607565, holotype	ON008483	—	Noordeloos et al. (2022b)
*E.dislocatum*	Spain	SFC-080612-01	ON008484	—	Noordeloos et al. (2022b)
*E.dislocatum*	Italy	TUF105920, paratype	UDB0799300	—	UNITE
*E.exile*	Germany	Lueck8	KP965773	KP965791	Karich et al. (2015)
*E.exile*	–	KM187354	MF977976	—	Unpublished in GenBank
*E.griseocyaneum*	Russia	LE254351	KC898444	KC898498	Morozova et al. (2014)
*E.griseocyaneum*	Germany	KaiR997	MZ611684	—	Reschke et al. (2022b)
*E.icarus*	Vietnam	LE F-312696, holotype	OM987257	OM996174	Morozova et al. (2022)
*E.icarus*	Vietnam	LE F-312697	OM987258	—	Morozova et al. (2022)
*E.incanum*	Sweden	LE312503, neotype	OK161247	OK161275	Crous et al. (2021b)
*E.incanum*	Russia	LE311794	OK161249	OK161276	Crous et al. (2021b)
*E.incanum*	Russia	LE315858	OK161250	—	Crous et al. (2021b)
*E.isborscanum*	Russia	LE312486	MW934564	—	Crous et al. (2021a)
*E.isborscanum*	Russia	LE302088, holotype	MW934566	MW934253	Crous et al. (2021a)
*E.linkii*	Norway	O-F-256353	UDB07673651	—	UNITE
*E.mastoideum*	China	GDGM28820	JQ281476	JQ410328	He et al. (2012)
*E.mastoideum*	China	GDGM26597	JQ291564	JQ320126	He et al. (2012)
*E.meridionale*	Greece	ACAM2014-0127	OL679698	—	Lebeuf et al. (2021)
*E.meridionale*	Greece	ACAM2018-0152	OL679699	—	Lebeuf et al. (2021)
*E.meridionale*	Greece	ACAM2018-0153, holotype	OL679700	—	Lebeuf et al. (2021)
*E.minutigranulosum*	Russia	LE312484	MZ145210	—	Dima et al. (2021)
*E.minutigranulosum*	Russia	LE312483	MZ145212	—	Dima et al. (2021)
*E.minutigranulosum*	Russia	LE302096, holotype	MZ145214	—	Dima et al. (2021)
*E.mougeotii*	Estonia	TUF106917	UDB015645	—	UNITE
*E.mougeotii*	Estonia	TUF101633	UDB016265	—	UNITE
*E.mougeotii*	Estonia	TUF106505	UDB019720	—	UNITE
*E.mutabilipes*	Finland	TUR610/12	LN850550	—	Kokkonen (2015)
*E.mutabilipes*	Estonia	TUR8788	LN850551	—	Kokkonen (2015)
*E.notabile*	Cyprus	L-0607514, holotype	OL343537	—	Vila et al. (2021)
*E.olivaceomarginatum*	USA	PUL00036174	ON561593	—	Unpublished in GenBank
***E.orientosinense***	**China**	**HFJAU1414, holotype**	**PQ584686**	—	**This work**
***E.orientosinense***	**China**	**HFJAU1907**	**PQ584690**	**PQ584707**	**This work**
***E.orientosinense***	**China**	**HFJAU2616**	**PQ584687**	—	**This work**
***E.orientosinense***	**China**	**HFJAU2920**	**PQ584688**	**PQ584708**	**This work**
***E.orientosinense***	**China**	**HFJAU4048**	**PQ584689**	**PQ584709**	**This work**
*E.pallidostriatum*	Spain	L-0607566, holotype	NR_177630	—	Vila et al. (2021)
*E.perasprellum*	France	GC01100310, holotype	MZ145177	—	Dima et al. (2021)
*E.perasprellum*	Sweden	GB-0204547 / JBJ 19-107	MZ145179	—	Dima et al. (2021)
*E.perasprellum*	Sweden	GB-0204548 / JBJ 19-122	MZ145180	—	Dima et al. (2021)
*E.perchalybeum*	Sweden	GB-0209474, holotype	NR_182490	—	Noordeloos et al. (2022b)
*E.perchalybeum*	Finland	TUR190180	ON008495	—	Noordeloos et al. (2022b)
*E.poliopus*	Estonia	TUF120264	UDB024655	—	UNITE
*E.praegracile*	China	GDGM29251	JQ281482	JQ320129	He et al. (2013)
*E.praegracile*	China	GDGM29256	JQ320107	—	He et al. (2013)
*E.pseudocoelestinum*	Germany	Lueck10	KP965774	KP965792	Karich et al. (2015)
*E.pseudocoelestinum*	–	KM132400	MF977966	—	Unpublished in GenBank
*E.pseudosubcorvinum*	Thailand	SDBR-CMUNK0985, holotype	MZ215769	MZ203540	Bhunjun et al. (2022)
*E.pseudosubcorvinum*	Thailand	SDBR-CMUNK1367	MZ215770	MZ203541	Bhunjun et al. (2022)
*E.pulchripes*	Russia	LE312485	MZ145187	—	Dima et al. (2021)
*E.pulchripes*	Russia	LE311808, holotype	MZ145188	—	Dima et al. (2021)
*E.pulchripes*	Russia	LE311809	MZ145189	—	Dima et al. (2021)
*E.queletii*	Turkey	OKA-TR1002	MT741747	—	Unpublished in GenBank
*E.queletii*	Estonia	TUF141044	UDB07674927	—	UNITE
*E.riparium*	Italy	L-0607563, holotype	NR_177632	—	Vila et al. (2021)
*E.riparium*	Estonia	TUF120259	UDB024650	—	UNITE
*E.septentrionale*	Norway	O-F-254295, holotype	NR_174647	—	Noordeloos et al. (2021)
*E.sericeum*	Germany	KaiR237	OL338118	OL338542	Reschke et al. (2022a)
*E.sericeum*	–	VHAs03 2	DQ367430	DQ367423	Unpublished in GenBank
*E.serrulatum*	Norway	O-F-158208/DMS-730296	MZ869016	—	Reschke et al. (2022b)
*E.serrulatum*	Russia	LE254361	KC898447	KC898501	Morozova et al. (2014)
*E.serrulatum*	Iran	EnSe-1	KT833862	—	Unpublished in GenBank
*E.sicoense*	Portugal	PO F2244, holotype	OR026624	—	Fachada et al. (2023)
*E.sicoense*	Portugal	PO F2245	OR026625	—	Fachada et al. (2023)
*E.subcaesiocinctum*	China	SAAS103	KY711235	KY972698	He et al. (2017)
*E.subcaesiocinctum*	China	SAAS133, holotype	KY711236	KY972697	He et al. (2017)
*E.subcorvinum*	USA	MGW1494	KY744168	—	Unpublished in GenBank
*E.subcorvinum*	USA	SAT1518905	KY744169	—	Unpublished in GenBank
***E.subgriseosquamulosum***	**China**	**HFJAU3967**	**PQ584696**	—	**This work**
***E.subgriseosquamulosum***	**China**	**HFJAU3969, holotype**	**PQ584697**	**PQ584721**	**This work**
***E.subpraegracile***	**China**	**HFJAU1822, holotype**	**PQ584698**	**PQ584710**	**This work**
***E.subpraegracile***	**China**	**HFJAU3094**	**PQ584706**	**PQ584711**	**This work**
***E.subpraegracile***	**China**	**HFJAU3164**	**PQ584700**	**PQ584712**	**This work**
***E.subpraegracile***	**China**	**HFJAU3168**	**PQ584705**	—	**This work**
***E.subpraegracile***	**China**	**HFJAU5110**	**PQ584701**	—	**This work**
***E.subpraegracile***	**China**	**HFJAU5115**	**PQ584699**	**PQ584713**	**This work**
***E.subpraegracile***	**China**	**HFJAU5140**	**PQ584702**	**PQ584714**	**This work**
***E.subpraegracile***	**China**	**HFJAU5175**	**PQ584703**	**PQ584715**	**This work**
***E.subpraegracile***	**China**	**HFJAU5177**	**PQ584704**	**PQ584716**	**This work**
*E.subserrulatum*	USA	TENN:068464	KY744143	—	Unpublished in GenBank
*E.subserrulatum*	USA	TENN:070407	KY744177	—	Unpublished in GenBank
*E.subtenuicystidiatum*	China	GDGM 28459, holotype	JQ320109	JQ320116	He et al. (2013)
*E.subtenuicystidiatum*	China	GDGM 29246	JQ320114	JQ320132	He et al. (2013)
*E.turci*	Austria	WU25055	UDB0802163	—	UNITE
*E.viridomarginatum*	The Netherlands	JAC15761	MW775255	—	Unpublished in GenBank
*E.viridomarginatum*	The Netherlands	JAC12344	MW775264	—	Unpublished in GenBank
***E.wuyishanense***	**China**	**HFJAU3571, holotype**	**PQ584691**	—	**This work**
***E.wuyishanense***	**China**	**HFJAU3871**	**PQ584692**	**PQ584717**	**This work**
***E.wuyishanense***	**China**	**HFJAU3874**	**PQ584694**	**PQ584718**	**This work**
***E.wuyishanense***	**China**	**HFJAU3878**	**PQ584693**	**PQ584719**	**This work**
***E.wuyishanense***	**China**	**HFJAU3881**	**PQ584695**	**PQ584720**	**This work**

## ﻿Results

### ﻿Phylogenetic analysis

A total of 2136 characters were used in subsequent analyses (ITS, 841 bp; LSU, 1,295 bp), of which 1382 were constant, 670 were parsimony-informative, and 84 were singleton. For Bayesian analysis, the average standard deviation of split frequencies was less than 0.01 after 20 million generations.

**Figure 1.**
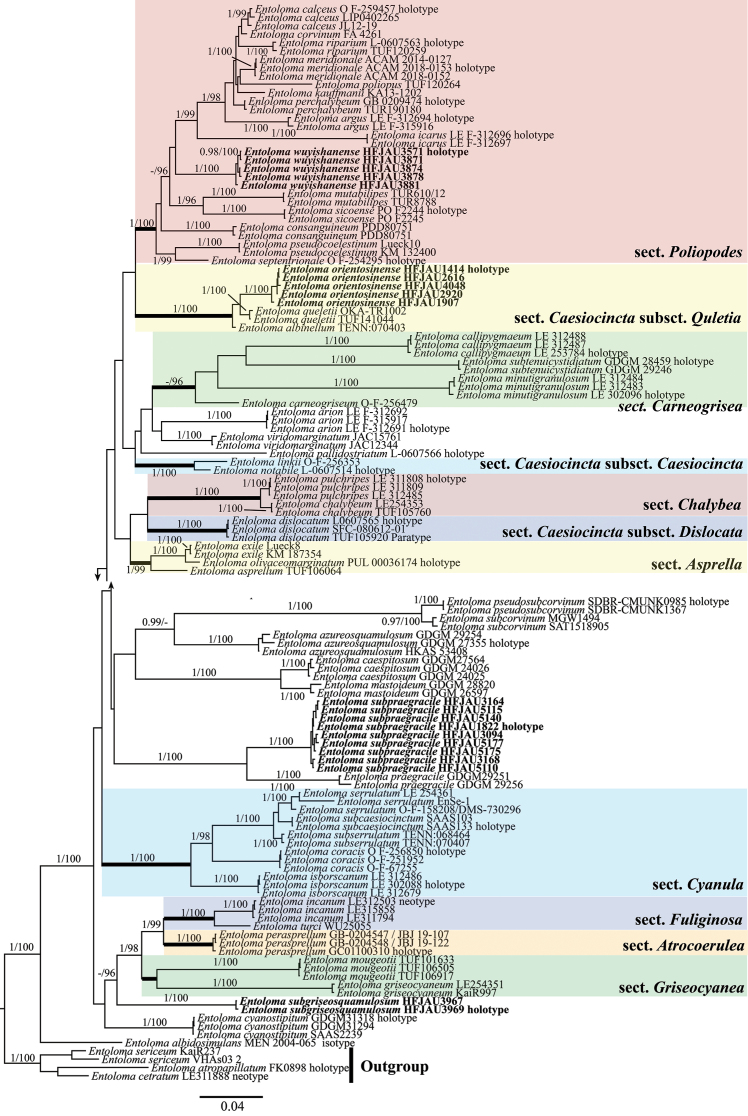
Phylogram of Entolomasubg.Cyanula spp. generated by Bayesian inference (BI) analysis based on ITS and LSU, rooted with E.subgenusNolanea spp. Bayesian inference (BI-PP) ≥ 0.95 and ML bootstrap proportions (ML-BP) ≥ 95% are indicated as PP/BP. The new taxa are marked in bold.

The results of the phylogenetic analysis were shown in Fig. 1. The results were consistent with previous studies (Noordeloos et al. 2022a; Brandrud et al. 2023). The four new species were clustered in the subg. Cyanula clade and formed separate and well-supported branches, respectively. Among them, *Entolomaorientosinense* formed a separate and well-supported lineage (BI-PP = 1, ML-BP = 99%), and groups together with *E.albinellum* (Peck) Hesler and *E.queletii* (Boud.) Noordel. nested in the sect. Caesiocincta, subsect. Queletia (BI-PP = 1, ML-BP = 100%). *Entolomasubgriseosquamulosum* independently formed a well-supported branch (BI-PP = 1, ML-BP = 100%). *Entolomasubpraegracile* formed a sister lineage with *E.praegracile* Xiao L. He & T.H. Li (BI-PP = 1, ML-BP = 100%), well clustered in a small clade (BI-PP = 1, ML-BP = 100%). *Entolomawuyishanense* formed a well-supported lineage within the sect. Poliopodes (BI-PP = 1, ML-BP = 100%).

### ﻿Taxonomy

#### ﻿ Entoloma orientosinense

Taxon classificationFungiAgaricalesEntolomataceae

J.Q. Yan, L.G. Chen & S.N. Wang sp. nov.

5938C910-C86F-59F2-84B5-A5E6EA5161B7

858361

Fig. 2

##### Etymology.

Refers to its type specimen originating from the eastern regions of China.

##### Holotype.

China • Anhui Province, Chizhou City, Shitan County, Guniujiang Nature Reserve, 30.0303°N, 117.5290°E, alt. 783 m, 9 October 2019, collected by Yu-Peng Ge, HFJAU1414.

##### Diagnosis.

*Entolomaorientosinense* is mainly characterized by the white, collybioid basidiomata, fibrillous and not striate pileus, narrow, adnate to decurrent lamellae, glabrous stipe, 5–6 angled basidiospores, sterile lamellae edge of carneogriseum-type, cylindrical to subclavate cheilocystidia, absence of cell pigments and clamp connections in hyphae. It differs from *E.albinellum* by its non-striate pileus, adnate to decurrent lamellae, and smaller basidiospores.

##### Macromorphology.

Basidiomata rather small, collybioid. Pileus 8–20 mm wide, convex then flattened with depressed center, with entire margin, slightly hygrophanous, fibrillous when young, then repent or raised scaly, not translucently striate, white (3A1–2). Lamellae moderately distant, 1.5–2.0 mm wide, with three types of lamellulae, adnate to decurrent, subventricose, initially white, then pink (11B4–6), with serrulate and concolourous edge. Stipe 20–25 × 2.0–3.0 mm, central, terete, tapered upwards, hollow, concolorous or paler with the pileus, minutely tomentose in the upper part elsewhere smooth and glabrous, base with white tomentum. Context thin, concolorous to the surface. Odor indistinct, taste not tested.

##### Micromorphology.

Basidiospores (8.5)9.3–11.0(12.5) × (6.0)6.5–8.0(9.0) μm, (av = 10.1 ×7.3 μm), Q = 1.2–1.6(1.7) (Qm = 1.4 ± 0.07, n = 200), heterodiametrical, 5–6 angles in profile view, thick-walled, inamyloid. Basidia 40–52 × 11–13 μm, clavate, 4-spored, sterigmata 5.0–10 μm long, clampless. Pleurocystidia absent. Lamellae edge sterile of carneogriseum-type. Cheilocystidia regularly dispersed in the lamellae edge, 17–47 × 4.0–7.0 μm, narrowly cylindrical to subclavate, septate, with slightly inflated apex. Lamellar trama regular, made up of cylindrical hyphae 7.0–13 µm wide. Pileipellis a cutis made up of cylindrical hyphae 8.0–11 μm broad, with transitions to a trichoderm towards the margin with clavate terminal elements 10–18 μm wide, not pigmented. Stipitipellis a cutis composed of densely arranged, cylindrical hyphae, up to 11 μm wide, slightly constricted at the septa, with acute or tapered end. Clamp connections absent.

**Figure 2.**
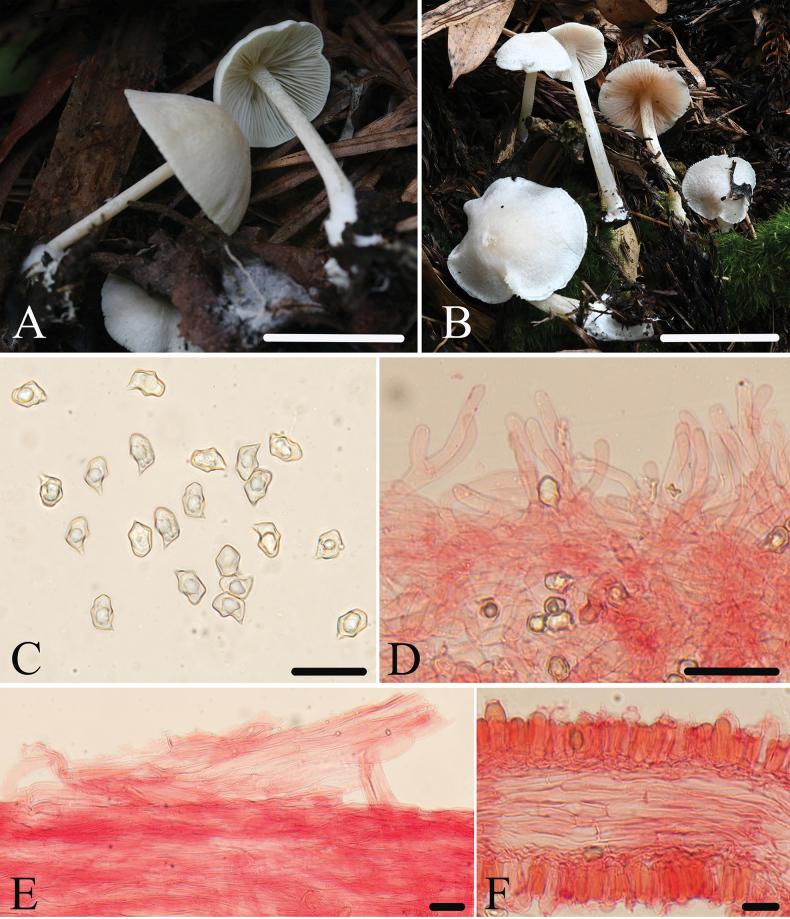
*E.orientosinense***A, B** basidiomata **A** HFJAU1414, holotype **B** HFJAU2616 **C** basidiospores **D** cheilocystidia **E** pileipellis **F** lamellar trama. All microscopic structures were observed in 5% KOH, and used 1% Congo red as the stain except **C**. Scale bars: 10 mm (**A, B**); 20 μm (**C**); 30 μm (**D–F**).

##### Habitat.

Solitary or scattered on soil in mixed coniferous-broad-leaved forest, or on rotten wood, soil, and moss in broadleaved forest.

##### Distribution.

So far known from eastern China.

##### Additional specimens examined.

China • Fujian Province, Wuyishan City, 27.7139°N, 117.6533°E, alt. 1113 m, 27 June 2022, collected by Jun-Qing Yan and Bing-Ring Ke, HFJAU4048 • Zhejiang Province, Lishui City, Suichang County, Huangtakou Village, 28.2679°N, 118.9435°E, alt. 346 m, 12 July 2020, collected by Jun-Qing Yan and Yan-Liu Chen, HFJAU1907 • Qingtian County, Shigu Lake, 28.2063°N, 120.0415°E, alt. 1130 m, 31 July 2021, collected by Jun-Qing Yan, Bing-Ring Ke, and Zhi-Heng Zeng, HFJAU2616 • Nanyang Village, 27.9603°N, 120.0020°E, alt. 522 m, 6 August 2021, collected by Yu-Peng Ge and Lan-Yu Sun, HFJAU2920.

##### Notes.

Morphologically, *Entolomaorientosinense* has much in common with *E.albidosimulans* G.M. Gates & Noordel. and *E.albinellum* with regard to the white and collybioid basidiomata. However, *E.albidosimulans* is distinct by its broader (up to 6 mm), adnate-emarginate lamellae, and belonging to E.subg.Alboleptonia species (Gates and Noordeloos 2007). *Entolomaalbinellum* differs from new species by its striate pileus, adnexed lamellae, and larger basidiospores (11–12.5 × 7.5–8.5 μm) (Hesler 1967).

In the molecular data, *E.orientosinense* fits well within subg. Cyanula, sect. Caesiocincta, subsect. Queletia including *E.albinellum* and *E.queletii*. *E.queletii* is distinguished from new species by the vinaceous-pink pileus and larger basidiospores (10–13 × 6.5–9.0 μm) (Bas et al. 1988a).

#### ﻿ Entoloma subgriseosquamulosum

Taxon classificationFungiAgaricalesEntolomataceae

J.Q. Yan, L.G. Chen & S.N. Wang sp. nov.

67316B68-5F59-5D0D-A406-72C02D4C85C9

858362

Fig. 3

##### Etymology.

Refers to its morphology similar to “*Entoloma griseosquamulosum*”.

##### Holotype.

China • Fujian Province, Wuyishan City, Yangzhuang Town, Xiyuan Village, 27.7632°N, 117.8139°E, alt. 533 m, 26 June 2022, collected by Jun-Qing Yan, Cheng-Feng Nie, and Lin-Gen Chen, HFJAU3969.

##### Diagnosis.

*Entolomasubgriseosquamulosum* is mainly characterized by the rather small, collybioid basidiomata, fuscous pileus, crowded and adnate lamellae, glabrous stipe, medium-sized basidiospores with 5–6 angles, mostly 5 angles, and absence of clamp connections. It differs from *E.griseosquamulosum* G.M. Gates & Noordel. by its gray stipe, smaller basidiospores, and absence of brilliant granules in hyphae.

##### Macromorphology.

Basidiomata rather small, collybioid. Pileus 11–20 mm wide, campanulate to convex with slight depressed center, with entire margin, not hygrophanous, gray hairy scaly with denser center, translucently striate almost up to 1/2 of the radius, fuscous (4D2–4F2) to dark gray (1F1–4F1), darker at center. Lamellae relatively crowded, 2.0–4.0 mm wide, with two types of lamellulae, adnate to emarginate, ventricose, initially white, then brownish-rose, with entire and concolorous edge. Stipe 20–42 × 1.5–3.0 mm, central, terete, equal, hollow, gray (1C1–1E1), darker downwards, sparsely white fibrillous in the upper part elsewhere smooth and glabrous, base with white mycelium. Context thin, white. Odor indistinct, taste not tested.

**Figure 3.**
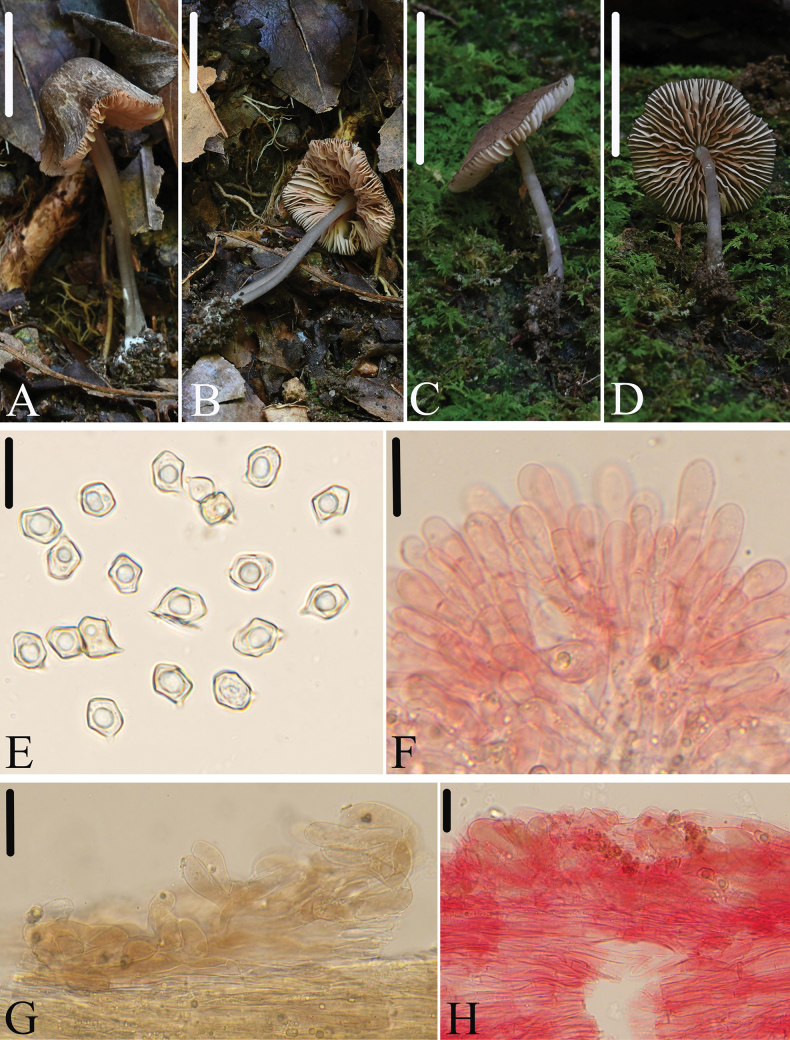
*E.subgriseosquamulosum***A–D** basidiomata **A, B** HFJAU3969, holotype **C, D** HFJAU3967 **E** basidiospores **F** cheilocystidia **G** basidia **G, H** pileipellis. **G** was observed in H_2_O, remaining microstructures all were observed in 5% KOH, and used 1% Congo red as the stain except **E**. Scale bars: 10 mm (**A–D**); 20 μm (**E**); 30 μm (**F–H**).

##### Micromorphology.

Basidiospores (8.1)8.4–10.5(11) × (6.0)6.5–8.0(8.5) μm, (av = 9.3 ×7.4 μm), Q = 1.1–1.4(1.5) (Qm = 1.3 ± 0.07, n = 100), subisodiameterical or heterodiametrical, 5–6 angles, mostly 5 angles in profile view, thick-walled, inamyloid. Basidia 27–36 × 10–13 μm, clavate, 4-spored, sterigmata 6.0–9.0 μm long, clampless. Pleurocystidia absent. Lamellae edge sterile of poliopus-type. Cheilocystidia 27–64 × 9.0–14 μm, clavate. Lamellar trama regular, made up of cylindrical hyphae 7.0–12 µm wide. Pileipellis a trichoderm made up of cylindrical hyphae 6.0–12 μm broad, with clavate terminal elements and yellow-brown intracellular pigment. Stipitipellis a cutis composed of densely arranged, cylindrical hyphae, 7.0–17 μm wide, slightly constricted at the septa, with acute or attenuated end. Clamp connections absent.

##### Habitat.

Solitary on soil or moss in broad-leaved forest.

##### Distribution.

So far known from Fujian Province in China.

##### Additional specimens examined.

China • Fujian Province, Wuyishan City, Yangzhuang Town, Xiyuan Village, 27.7652°N, 117.8164°E, alt. 512 m, 26 June 2022, collected by Jun-Qing Yan, Cheng-Feng Nie, and Lin-Gen Chen, HFJAU3967.

##### Notes.

Morphologically, several similar species within Entolomasubg.Cyanula that share brown to brown-gray pileus can be distinguished from the new species as follows: *E.anatinum* (Lasch) Donk is characterized by its larger basidiospores (9.0–13.5 × 7.5–9.0 μm) with 6–9 angles, and fertile lamellae edge (Donk 1949); *E.glaucobasis* Huijsman ex Noordel. has larger basidiospores (10–13.5 × 7.0–8.0 μm) (Noordeloos 1985); *E.griseosquamulosum* differs from the new species by the gray-violet stipe, larger basidiospores (9.0–12 × 7.0–9.0 μm), and presence of abundant brilliant granules in all hyphae (Noordeloos and Gates 2009); *E.phaeomarginatum* E. Horak is recognized by the fibrillose pileus, brown lamellae edge, and larger basidiospores (10–13 × 7.0–8.0 μm) (Horak 1973); *E.saponicum* G.M. Gates & Noordel. is distinct by the blackish brown lamellae edge and presence of abundant brilliant granules in all hyphae (Noordeloos and Gates 2009).

Phylogenetically, *E.cyanostipitum* Xiao L. He & W.H. Peng is closest to the new species. However, *E.cyanostipitum* is distinct by the deep blue pileus margin, lamellae edge and stipe, and the ITS region, with an 84% similarity (He et al. 2017).

#### ﻿ Entoloma subpraegracile

Taxon classificationFungiAgaricalesEntolomataceae

J.Q. Yan, L.G. Chen & S.N. Wang sp. nov.

5ABCDC59-6C3D-5F2A-B0B9-8DF005F732AE

856754

Fig. 4

##### Etymology.

Refers to its macroscopic morphology similar to “*Entoloma praegracile*”

##### Holotype.

China • Zhejiang Province, Lishui City, Qingyuan County, Bandaihoushang Village, 27.6748°N, 119.0780°E, alt. 1084 m, 7 July 2020, collected by Jun-Qing Yan and Yan-Liu Chen, HFJAU1822.

##### Diagnosis.

*Entolomasubpraegracile* is mainly characterized by the yellow, glabrous, and striate pileus, white, adnexed to adnate lamellae with tiny lateral veins, 5–7 angled and medium-sized basidiospores, sterile or heterogeneous lamellae edge of serrulatum-type, cylindrical or clavate cheilocystidia, and absence of clamp connections. It differs from *E.praegracile* by the larger basidiomata, and sterile or heterogeneous lamellae edge.

##### Macromorphology.

Basidiomata rather small. Pileus 10–20 mm wide, conical when young, then convex to flattened with depressed, rarely cuspidate center, with entire margin, not hygrophanous, smooth and glabrous, translucently striate almost up to the center, ochre (7B4–6), grayish yellow (1A4–5) to tawny (2C4–6), darker at center. Lamellae relatively dense, 1.5–2.0 mm wide, with tiny lateral veins and two or three types of lamellulae, adnate to adnexed, subventricose, white, with entire and concolorous edge. Stipe 25–35 × 2.0–2.5 mm, central, terete. equal, hollow, concolorous or paler with the pileus, smooth and glabrous, sometimes grooved, white tomentose at the base. Context thin, concolorous to the surface. Odor indistinct, taste not tested.

**Figure 4.**
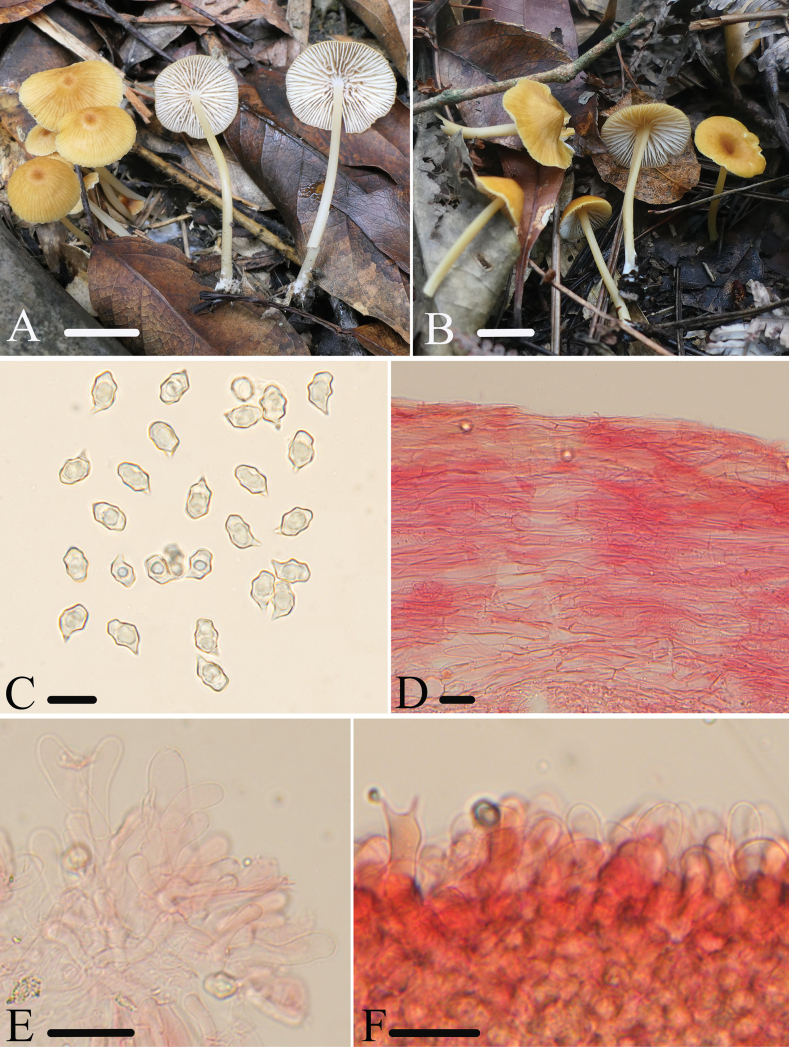
*E.subpraegracile***A, B** basidiomata **A** HFJAU1822, holotype **B** HFJAU5115 **C** basidiospores **D** pileipellis **E** cheilocystidia **F** heterogeneous lamellae edge. All microscopic structures were observed in 5% KOH, and used 1% Congo red as the stain except **C**. Scale bars: 10 mm (**A, B**); 20 μm (**C**); 30 μm (**D–F**).

##### Micromorphology.

Basidiospores (7.0)8.5–10.5(12) × (6.0)6.5–7.5(8.5) μm, (av = 9.6 ×7.0 μm), Q = 1.2–1.6(1.7) (Qm = 1.4 ± 0.08, n = 200), heterodiametrical, 5–7(8) angles in profile view, appearing nodulose, thick-walled, inamyloid. Basidia 27–37 × 9–12 μm, clavate, slightly constricted at middle, mainly 2-spored, sterigmata 6.0–12 μm long, clampless. Pleurocystidia absent. Lamellae edge sterile or heterogeneous of poliopus-type. Cheilocystidia dense clusters on lamellae edge, 21–53 × 7.0–14 μm, cylindrical or clavate. Lamellar trama regular, made up of cylindrical hyphae 4.0–8.0 µm wide. Pileipellis a cutis made up of cylindrical hyphae 5.0–12 μm broad, with transitions to a trichoderm towards the center with clavate terminal elements 10–16 μm wide, with tawny intracellular pigment. Stipitipellis a cutis composed of densely arranged, cylindrical hyphae, 7.0–15 μm wide, slightly constricted at the septa, with rounded end. Clamp connections absent.

##### Habitat.

Solitary or scattered on soil in mixed coniferous-broad-leaved forest.

##### Distribution.

So far known from eastern China.

##### Additional specimens examined.

China • Fujian Province, Wuyishan City, 27.8594°N, 117.9096°E, alt. 372 m, 12 August 2021, collected by Jun-Qing Yan and Ze-Wei Liu, HFJAU3094 • 27.8563°N, 117.8661°E, alt. 668 m, 13 August 2021, collected by Qin Na, Yu-Peng Ge, and Lan-Yu Sun, HFJAU3164, HFJAU3168 • 27.7221°N, 117.7072°E, alt. 654 m, 16 August 2023, collected by Nian-Kai Zeng, Cheng-Feng Nie, Hua-Zhi Qin, Hui Deng, Tian Jiang, and Run-Xiang Zhao, HFJAU5110, HFJAU5115, HFJAU5140, HFJAU5175, HFJAU5177.

##### Notes.

In the phylogenetic tree, *E.subpraegracile* groups together with *E.praegracile*. *Entolomapraegracile* differs from the new species by the smaller pileus (less than 10 mm), fertile lamellae edge, and the ITS sequence with 86% similarity (He et al. 2011).

Some similar species with a yellow pileus within subg. Cyanula can be distinguished from the new species as follows: *E.chloropolium* (Fr.) M.M. Moser is recognized by the fertile to heterogeneous lamellae edge, and septate cheilocystidia (Noordeloos 2004); *E.formosum* (Fr.) Noordel. is characterized by its squamulose pileus, larger basidiospores (9.0–12.5 × 6.0–8.0 μm), and fertile or heterogeneous lamellae edge (Bas et al. 1988b). *E.luteoochraceum* Ribes & Vila is distinct by the squamous pileus, 4-spored basidia, and fertile lamellae edge (Ribes and Vila 2013); *E.pseudoturci* Noordel. has tomentose to squamous and not striate pileus, porphyrogriseum-type lamellae edge, and brilliant granules in tissue cells (Noordeloos 1984).

#### ﻿ Entoloma wuyishanense

Taxon classificationFungiAgaricalesEntolomataceae

J.Q. Yan, L.G. Chen & S.N. Wang sp. nov.

23715415-FC9C-5C75-B4BC-5C7ADC9CD887

858363

Fig. 5

##### Etymology.

Refers to the collection locality of the holotype specimen – Wuyishan National Natural Park.

##### Holotype.

China • Fujian Province, Nanping City, Wuyishan National Natural Park, 27.5418°N, 117.4743°E, alt. 422 m, 7 June 2022, collected by Jun-Qing Yan and Lin-Gen Chen, HFJAU3571.

##### Diagnosis.

*Entolomawuyishanense* is mainly characterized by the rather small and blue basidiomata, squamous and striate pileus, white and adnexed lamellae with fertile edge with slightly bluish pigmentation near the stipe, relatively large basidiospores with 5–6 angles, pileipellis with fuscous intracellular pigment. It differs from *E.azureosquamulosum* Xiao L. He & T.H. Li by the striate pileus, adnexed lamellae, larger basidiospores, and fertile lamellae edge.

**Figure 5.**
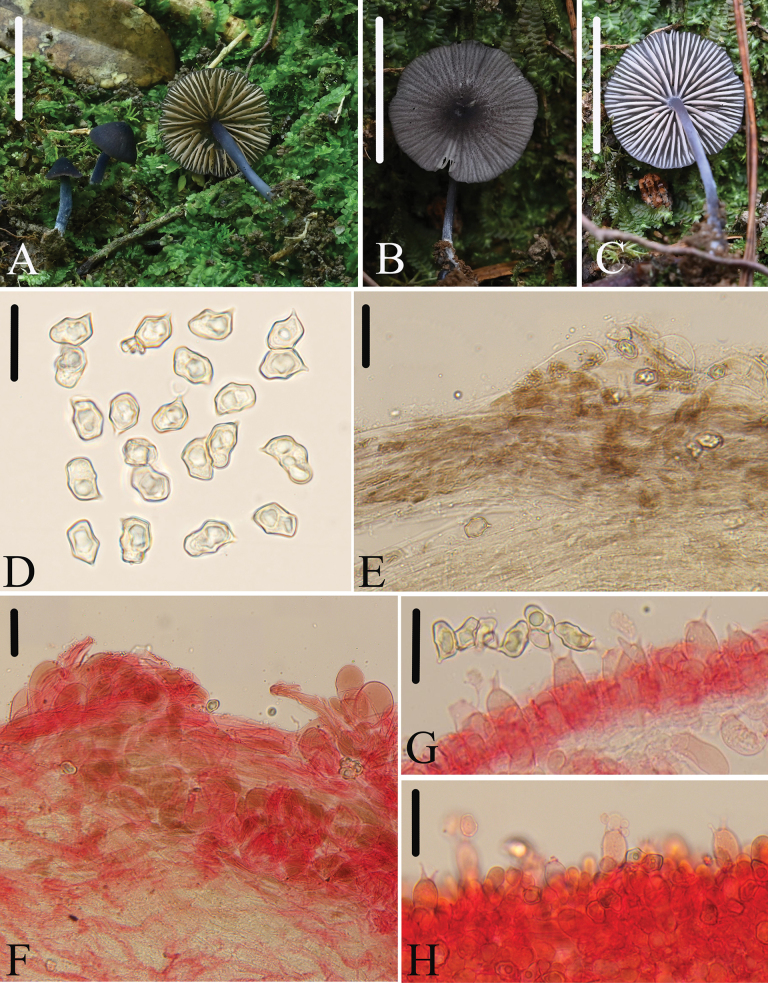
*E.wuyishanense***A–C** basidiomata HFJAU3571, holotype **B, C** HFJAU3871 **D** basidiospores **E, F** pileipellis **G** basidia **H** fertile lamellar edge. **E** was observed in H_2_O **D, F–H** were observed in 5% KOH, and used 1% Congo red as the stain except **D**. Scale bars: 10 mm (**A–C**); 20 μm (**D**); 30 μm (**E–H**).

##### Macromorphology.

Basidiomata rather small. Pileus 2.0–11 mm wide, conical when young, then convex to flattened with depressed center, with entire, straight or wavy margin, not hygrophanous, squamous with denser center, translucently striate almost up to the center, deep blue (20E4–7) to light gray-blue (20B2–3), darker at center. Lamellae moderately distant, 1.0–3.0 mm wide, with two types of lamellulae, adnexed, ventricose, white, with entire and bluish edge near the stipe. Stipe 9.0–26 × 1.0–2.0 mm, central, terete, equal, hollow, concolorous with pileus, paler downwards, white fibrillose, glabrescent with age, base with white mycelium. Context thin, gray-blue. Odor indistinct, taste not tested.

##### Micromorphology.

Basidiospores (9.5)10–13.5(15) × (6.5)7.5–9.5(10) μm, (av = 11.7 ×8.5 μm), Q = 1.2–1.7(2.0) (Qm = 1.4 ± 0.11, n = 200), heterodiametrical, 5–6 angles in profile view, sometimes appearing nodulose, thick-walled, inamyloid. Basidia 25–33 × 10–13 μm, clavate, slightly constricted at middle, 4- or 2-spored, sterigmata 6.0–10 μm long, clampless. Lamellae edge fertile. Cystidia absent. Lamellar trama regular, made up of cylindrical hyphae 5.0–11 µm wide. Pileipellis a trichoderm made up of clavate to pyriform terminal cells, 41–65 × 22–36 μm. Pigment fuscous, intracellular, diffuse in pileipellis. Stipitipellis a cutis composed of densely arranged, cylindrical hyphae, 5.0–12 μm wide, with rounded end. Clamp connections absent.

##### Habitat.

Solitary or scattered on moss in mixed coniferous-broad-leaved forest.

##### Distribution.

So far known from eastern China.

##### Additional specimens examined.

China • Zhejiang Province, Lishui City, Songyang County, Zicao Village, 28.4874°N, 119.5783°E, alt. 722 m, 2 July 2022, collected by Jun-Qing Yan, Cheng-Feng Nie, and Meng-Hui Han, HFJAU3871, HFJAU3874, HFJAU3878 • Lishui City, Yunhe County, Chongtou Town, Xiayang Village, 28.0499°N, 119.4732°E, alt. 592 m, 4 July 2022, collected by Jun-Qing Yan and Cheng-Feng Nie, HFJAU3881.

##### Notes.

Morphologically, *E.azureosquamulosum* is the most similar species with the distinction that *E.azureosquamulosum* exhibits not striate pileus, adnate lamellae, smaller basidiospores (8–10.5 × 6.5–8.0 μm), and sterile lamellae edge (He et al. 2012).

In the phylogenetic tree, *E.wuyishanense* belongs to Cyanulasect.Poliopodes, within which several species have blue pileus, including *E.argus* O.V. Morozova, E.S. Popov, A.V. Alexandrova & Noordel., *E.calceus* Noordel., Bendiksen, Brandrud, P.-A. Moreau & Vila, *E.corvinum* (Kühner) Noordel., *E.icarus* O.V. Morozova, E.S. Popov & Noordel., and *E.perchalybeum* Noordel., J.B. Jordal & Dima. However, the lamellae edge of the latter in all species is sterile. In addition, *E.argus* is characterized by the adnate lamellae and smaller basidiospores (≤10 μm) (Morozova et al. 2022); *E.calceus* shows 6–9 angled basidiospores (Noordeloos et al. 2022b); *E.corvinum* is recognized by its not striate pileus, adnate lamellae, and smaller basidiospores (8.0–11 × 6.5–7.5 μm) (Noordeloos 1982); *E.icarus* can be easily differentiated by the lateral stipe and adnate lamellae (Morozova et al. 2022); *E.perchalybeum* is distinct by the adnate lamellae and 6–7 rather bluntly angled basidiospores (Noordeloos et al. 2022b).

## ﻿Discussion

Entolomasubg.Cyanula currently is divided into 11 sections (Noordeloos et al. 2022a; Dima et al. 2023), and which formed well-supported clades and confirmed taxonomic positions within subgenus in this study. It is worth mentioning that two of the four newly discovered species in this study, along with the majority of previously reported new taxa of subg. Cyanula from China, do not belong to any known sections. To address this issue, continued phylogenetic studies of subg. Cyanula based on both morphological characters and molecular markers for more representative specimens of this subgenus from China are necessary. This will result in a more natural classification in the future.

Notably, based on the results of this phylogenetic analysis, we have realized that the sect. Caesiocincta is divided into three clades. However, since none of the originating branches of these scattered clades is supported, the cause of this result cannot be determined. Additional specimen data are needed for further analysis.

The present study expands our understanding of entolomoid species by providing descriptions and phylogenetic analyses for four new species. The findings enrich our knowledge of the distribution of E.subg.Cyanula species in China and the overall diversity of *Entoloma*.

### ﻿Key to Entolomasubg.Cyanula species reported in China

**Table:** 

1	Pileus white to pink	**2**
–	Pileus other colored	**3**
2	Pileus white, not striate, with depressed center; lamellae adnate to decurrent; basidiospores 5–6 angled; pigment not	***E.orientosinense***
–	Pileus pink, striate, with umbonate center; lamellae subfree to adnexed; basidiospores 6–8 angled; pigment yellow encrusting	***E.mastoideum***
3	Pileus yellow-brown to grayish-brown	**4**
–	Pileus blue to violaceous	**13**
4	Pileus glabrous to fibrillose	**5**
–	Pileus squamulose to velvety	**8**
5	Basidiospores Lav ≥ 11 μm; pileus striate, with depressed center; lamellae adnate; lamellae edge sterile or heterogeneous	***E.subtenuicystidiatum***
–	Basidiospores Lav < 11 μm	**6**
6	Pileus ≥ 20 mm, with margin exceeding lamellae; lamellae adnate-emarginate to adnexed	***E.caespitosum***
–	Pileus < 20 mm	**7**
7	Lamellae edge sterile or heterogeneous; pileus not hygrophanous	***E.subpraegracile***
–	Lamellae edge fertile; pileus hygrophanous	***E.praegracile***
8	Lamellae edge fertile; pileus striate; lamellae adnate or emarginate; basidiospores 8.0–14 × 5.5–10 μm	***E.insidiosum***
–	Lamellae edge sterile	**9**
9	Basidiospores Lav ≥ 10 μm; pileus striate; lamellae adnexed; pigment yellow-brown intracellular	***E.longistriatum***
–	Basidiospores Lav < 10 μm	**10**
10	Cheilocystidia cylindrical to clavate	**11**
–	Cheilocystidia fusiform, lageniform, vesiculose to spheropedunculate	**12**
11	Pileus striate; lamellae adnate to emarginate; lamellae edge entire and concolorous with lamellae	***E.subgriseosquamulosum***
–	Pileus not striate; lamellae adnexed to short decurrent; lamellae edge serrulate and blue-black	***E.subcaesiocinctum***
12	Cheilocystidia fusiform to lageniform; pileus not striate; lamellae adnexed to free; lamellae edge concolorous with lamellae	***E.pseudosubcorvinum***
–	Cheilocystidia vesiculose or spheropedunculate; pileus not striate; lamellae adnate-emarginate; lamellae edge brown	***E.pulchripes***
13	Lamellae edge fertile; pileus squamous, striate; lamellae adnexed; lamellae edge blue; basidiospores 10–13.5 × 7.5–9.5 μm	***E.wuyishanense***
–	Lamellae edge sterile	**14**
14	Pileus not striate	**15**
–	Pileus striate	**16**
15	Lamellae adnate-emarginate; cheilocystidia fusoid	***E.azureosquamulosum***
–	Lamellae short decurrent; cheilocystidia cylindrical to subclavate	***E.cyanostipitum***
16	Cheilocystidia subglobose or sphaeropedunculate; lamellae edge concolorous with lamellae	***E.ekaterinae***
–	Cheilocystidia broadly clavate or lageniform; lamellae edge blackish purple	***E.callipygmaeum***

## Supplementary Material

XML Treatment for Entoloma orientosinense

XML Treatment for Entoloma subgriseosquamulosum

XML Treatment for Entoloma subpraegracile

XML Treatment for Entoloma wuyishanense

## ﻿Additional information

### Author ORCIDs

Lin-Gen Chen https://orcid.org/0009-0000-2506-181X

Hong Chen https://orcid.org/0009-0004-0107-3962

Ling Ding https://orcid.org/0009-0000-5293-046X

Hui Zeng https://orcid.org/0000-0003-2025-844X

Sheng-Nan Wang https://orcid.org/0000-0003-0648-271X

Jun-Qing Yan https://orcid.org/0000-0003-1128-5171

### Data availability

All of the data that support the findings of this study are available in the main text or Supplementary Information.

## Supplementary materials

Supplementary material 1 Supplementary data Data type nex Explanation note A nexus file contains alignment sequence and original tree of ML and Bayes. https://binary.pensoft.net/file/1318709This dataset is made available aunder the Open Database License (http://opendatacommons.org/licenses/odbl/1.0/). The Open Database License (ODbL) is a license agreement intended to allow users to freely share, modify, and use this Dataset while maintaining this same freedom for others, provided that the original source and author(s) are credited.Lin-Gen Chen, Hong Chen, Ling Ding, Yu-Qin Xu, Hui Zeng, Sheng-Nan Wang, Jun-Qing Yan
